# Book Reviews

**Published:** 1876-06

**Authors:** 


### Book Reviews.

[NOTE. — All works reviewed in the pages of the CHICAGO MEDICAL JOURNAL AND EXAMINER may be found in the extensive stock of W. B. KEEN, COOKE & Co., whose catalogue of Medical Books will be sent to any address upon request.]

HOSPITAL PLANS. Five Essays relating to the Construction, Organization and Management of Hospitals, contributed by their authors for the use of the Johns Hopkins Hospital, of Baltimore. New York, 1875. William Wood & Co.

This is one of the most valuable books of the current season. It has its origin in the munificence of the late Johns Hopkins, of Baltimore, who bequeathed a sum of money, now amounting to three millions of dollars, for the establishment and maintenance of a hospital, an orphan asylum, a training school for nurses, and a medical college, in the city of Baltimore. Placing this great sum in the hands of twelve trustees, he instructed them, in order to "provide for a hospital, which shall, in construction and arrangement, compare favorably with any other" similar institution in the world, "to obtain the advice and assistance of those, at home and abroad, who have achieved the greatest success in the construction and management of hospitals." The trustees, accordingly, authorized their Building Committee "to confer with five distinguished physicians, * * * who have made hospitals their special study, and obtain from them such advice as they may need, and to compensate them for it." The five gentlemen thus signalized are Norton Folsom, M.D., Superintendent of the Massachusetts General Hospital, Prof. Stephen Smith, M.D., well known for his studies in behalf of the Roosevelt Hospital in New York, Caspar Morris, M.D., patron saint of the Episcopal Hospital in Philadelphia, Asst. Surgeon John S. Billings, of the Surgeon General's staff in Washington, and Joseph Jones, M.D., the celebrated Professor of Chemistry and Clinical Medicine, in the Medical Department of the University of Louisiana. Work thus inaugurated could not fail to be well done, and in this volume we have the five essays thus evoked.

These essays are read in Chicago with peculiar interest, because they contain in full, information concerning those questions of hospital construction which were recently investigated by a committee of the Medical Board of the Cook County Hospital—questions to which the answers thus elicited are now being embodied in our new County Hospital which is so rapidly approaching its completion. This committee, consisting of a physician, a surgeon and an architect, visited the cities of the East; inspected their hospitals; and there conferred with the authors of this book, as well as with other gentlemen who have made a special study of hospital construction. We flatter ourselves, consequently, that in this new Chicago hospital may be found all the excellences of older hospitals, with an absence of their defects.

In the report under consideration we learn that the site of the Johns Hopkins Hospital covers about fourteen acres. The Chicago site embraces nearly thirteen acres, but here the parallelism ends, for the Baltimore hospital will stand on elevated ground with an undulating surface, while our buildings are erected upon the flat prairie where it is only nine feet above the level of the lake. The essayists in this volume have, with the exception of Dr. Smith, touched lightly upon the subject of hospital location. Dr. S., however, presenting the results of Pettenkofer's studies concerning *ground air* and *ground water*, leaves little to be desired in this particular. He insists upon the importance of the most thorough drainage, to remove ground water, and to keep what cannot be removed at a uniform level, quoting the statement of Dr. Carpenter that when water rises in the soil, "typhoid and its allied diseases become prevalent, but as the water line falls * * *, ague, neuralgia, rheumatic disorders are rife." The use of porous tile-drains is recommended, to favor a more perfect withdrawal not only of water but of air from the soil, it being argued that the earth is generally charged with carbonic acid and other noxious gases. The ground air is also liable to become charged with the products of putrefaction when the yards are not properly policed; and it is also the great reservoir of such malarial germs as may be produced in the earth under the influence of heat and moisture—hence it can not be too carefully excluded from all buildings. Acting upon this conviction our new hospital is brought into the most intimate connection with the system of sewerage for the city, and the air which is supplied to the buildings is not allowed to enter at the level of the earth, but is drawn in at the top of towers fifteen feet high.

Having disposed of the question of location, our authors consider the character of the buildings best adapted for a hospital. All are unanimous in recommendation of a system of detached buildings, grouped around the kitchen, laundry, engine-house and mortuary, and connected by a covered way or corridor. There seems to be little difference of opinion regarding the general mode of construction of these important offices, and of the executive building. Dr. Folsom presents for a mortuary and for a surgical amphitheatre the most elaborate plans—which are in fact the plans of the Massachusetts General Hospital amphitheatre and mortuary, and which have been, with slight modification, adopted in the construction of the new Cook County Hospital. But, regarding the construction of the hospital-ward buildings, great difference of opinion appears among the essayists. The question of temporary versus permanent edifices is fully discussed, and is unanimously decided in favor of substantial structures of brick and stone. The notion of erecting a system of wooden huts for the reception of the sick of a great civic population has here received its quietus. In fact, one need only visit such an institution as the Cook County Alms House, where the poor and the sick are lodged in temporary wooden barracks, to see what would be the practical result if such a proposition were carried into effect.

Permanent edifices being the unanimous choice of the essayists, they proceed to discuss the question whether hospital wards should be aggregated in buildings of several stories, or whether they should be segregated in structures of only one story. Prof. Smith pronounces decidedly in favor of a single ward in each building. Prof. Jones obscures his opinion with a cloud of words. Dr. Folsom does not deny that patients get well in buildings of many stories, but is in favor of solitary wards. Dr. Billings believes that by a proper arrangement of stairways—an arrangement which has been introduced in our Chicago hospital—wards can be so isolated that buildings of several stories may be as healthful as buildings of only one story. To Doctor Morris, however, belongs the credit of having discussed this whole subject in the most enlightened and conclusive manner. He sums up the whole argument as follows:

"The experience not only of private dwellings but of public institutions, in which many persons are brought under one roof, is very strongly in favor of the superposition of one story upon another. Not only are the upper rooms more light, and cheerful, and airy, but they are also more healthy. Army experience confirms this also. In buildings provided for troops, even in miasmatic districts, those men who occupy the upper floors are less subject to disease than those on ground floors, which would not be the case if that malarious influence ascended; and travelers are aware that in foreign cities the upper floors of hotels are appropriated to the best apartments, those on the two lower floors being considered less healthy than those above. It is a well established fact that prisoners confined in upper stories enjoy better health than those on ground floor apartments." p. 184. The Medical Board of our own hospital arrived at the same conclusion after a careful consideration of all the circumstances connected with hospital construction in our bleak northern climate. Isolated wards may be well enough in the South, where Spanish moss drapes the live-oak with funereal gloom, or under the palm-trees and broad-leaved bananas of the tropics; but upon an open prairie, under the leaden skies of the chilly North, a "pavilion" of one story is a thing to be inspected, admired, and—carefully avoided.

A full discussion of the subject of heating and ventilation proves the truth of this conclusion. The arguments for single wards are as follows: higher ceilings, ridge ventilation, and experience gathered during the civil war. But lofty ceilings imply greater expense for fuel. Ridge ventilation adds to that expense, and insures a cold atmosphere in the lower stratum of the ward. If ridge ventilation is abandoned, and a proper system of aspiration by means of a central shaft is introduced, the ward will be warm and well ventilated, but every argument against the superposition of another ward will be removed. As for the experience during the civil war—results obtained from the treatment of vigorously constituted young men, in a temperate climate, in hospitals furnished and administered with a munificence such as the world had never before witnessed, when contrasted with the results of treatment of the dregs of civic populations, in buildings inherited from the "ages of faith," under conditions of administration of the most restricted character, can never be logically used to bolster up an argument in favor of the superiority of single story buildings. The only thing proved by such experience is the superiority of good construction and management when compared with bad arrangement, poor nursing, and scanty diet.

A great deal of space is occupied by the essayists with a consideration of the best method for heating and ventilation—these two functions being inseparably connected during the cold months of the year. All agree that no system of artificial ventilation can equal that obtained in summer through open windows, but that is out of the question in winter. Introduction of pure air, heated by contact with steam coils below the ward, through openings in the floor, and its discharge through the ceiling, is considered the most effectual method; but its cost is enormous. It can only be employed when expense is to be left out of consideration. A room can be thoroughly warmed and very well ventilated, at half the cost of the previous mode, by introduction of warm air near or through the ceiling, and by its withdrawal through openings in the floor which communicate by a system of radiating ducts under the floor, with a central shaft in which the air is warmed and propelled upward by the heat of an iron smoke-stack. In this way a complete aspiration of the entire ward may be provided for. The wards of our new County Hospital are thus heated and ventilated. A register under each bed withdraws the air, and sucks down a fresh supply from the upper part of the room. In each corner is an open fire-place. A space of three or four feet between the floor and the ceiling of the ward below gives ample room for the arrangement of the numerous air-ducts, and this space is itself, by an ingenious contrivance, connected with the central aspirating shaft, so that stagnation of air anywhere about the building seems to be an utter impossibility.

The location of the heating apparatus is still an open question. The use of hot water seems to be impracticable in this latitude, and the authors generally concur in recommending the use of steam coils in the basement of each building. Dr. Billings expresses himself in favor of "giving to each pavilion (building) its own heating and ventilating apparatus." This has been done in the buildings thus far erected for the Cook County Hospital; but it is doubtful whether the advantages claimed for the introduction of two huge boilers directly under the floor of a ward will not be more than neutralized by the noise, by the dirt and by the coal-dust which will fill the basement, and will find its way into the coil-chambers from which it will be drawn up through the air-ducts into the wards. It is also a question whether the basement of each building will not be needed for purposes of storage, to the exclusion of heating apparatus, which may then be concentrated in the central engine-house. It is in the basement, too, that the location of dungeons for the confinement of maniacs and drunkards is recommended.

Much more might be written concerning the minor details of construction, upon which our authors dwell, but our space will not allow anything beyond an allusion to the salient points of this interesting volume. Dr. Folsom's essay is especially rich in suggestions respecting such usually "unconsidered trifles" as the best method of constructing a window or a transom. He seems to have omitted nothing, and in the minutest particular shows himself a thoroughly finished master of hospital construction and administration. With regard to the management of such an institution all are agreed that the superintendent, though not to be charged in any way with the care of the patients, should have the education of a physician. Every physician is by no means qualified to superintend a hospital. That requires a rare assemblage of gifts, both natural and acquired. But no man is competent to intelligently superintend the administration of such a great machine who is not thoroughly educated, and practically acquainted with the facts of anatomy, physiology, and therapeutics, as well as with physics and chemistry ; for no other education can enable one to fully appreciate the vital necessity for the greatest possible perfection in all arrangements and appliances for the care of the sick. And since such men can not easily be induced to take a position from which they are liable to be, at any moment, displaced at the will of a continually changing body of superior officials, it is all important that some greater degree of stability than is at present customary, should be incorporated into the management of public charitable institutions.

With this brief and imperfect notice we must commend to our readers one of the most interesting and instructive volumes that has recently attracted our attention.

H. M. L.

PRINCIPLES OF HUMAN PHYSIOLOGY. By *Wm. B. Carpenter, M.D.*, F.R.S., F.G.S., F.L.S., etc. Edited by Henry Power, M.B., etc. Eighth edition. Philadelphia: Lindsay & Blakiston. 1876.

A glance at the index of this work, its comprehensive list of subjects and authors, would lead us to anticipate what a study of the text proves true, that the author has condensed into one volume the material for at least three. Indeed, it covers even more ground than is occupied by Flint's five volumes. Hence our first criticisms: The necessary abridgment of subjects and the unwieldy shape of the book. As a compendium of physiology in the relations of the science to histology and pathology, it certainly brings the subject, as a whole, up to the level of the latest research. This is especially true of the nervous system, and still more especially of the encephalon, both as regards its anatomy by Meynert and its functions—particularly the function of the peripheral layer of cortical substance of the hemispheres—the arguments as to the existence of motor centres in the same put forth by Ferrier, Hitzig, Hermann, Sanderson, and others; also the function of the sensory ganglia—the common seat for sensational and mental consciousness.

The function of the cord is not so well defined. It has long been considered on good authority, that cerebral memory is but a higher degree of spinal, while the researches of Dr. Hammond (whose authority on the nervous system is scarcely quoted by this author) seem fully to justify his conclusions, viz: That perception and volition are seated in the spinal cord, as well as in the cerebral ganglia; that the cord is not probably capable of *originating* mental influence independently of sensorial impressions—a condition of the brain also, till it has accumulated facts through the operation of the senses; that as memory is not an attribute of the mental influence evolved by the spinal cord, it requires, unlike the brain, a new impression, in order that mental force may be produced.

Thus more and more are we departing from the old idea that the brain is the principal nerve-centre of the system, and approaching to the new idea that gives to the cord the first position. Of course we refer to the physiological rather than the anatomical cord—the track of gray globules which extends beyond the spinal canal even to the sella turcica. The physiological experiments of Legallois should have found a place in this particular subject. Altogether, there is not enough said as to the cumulative or storing-up power of the nerve globule—wherever that organ, for we must consider it such, may be found.

The mechanism of respiration is not as well explained as it might be, nor is the idea made sufficiently prominent that the essence of the function is in the tissues themselves. The preface acknowledges that due consideration is not given to the influence of respiration upon the circulation—that point is clearly developed when the true mechanism is made plain. The function of the liver is recognized as double, viz., glycogenic and biliary, but we find nothing concerning that complicated point in the anatomy of the liver as to whether the structure is not also a double one—a question which arises from the study of embryonic development, the biliary gland being a development from the intestinal tube, while the glycogenic is an outgrowth from the omphalo-mesenteric or portal vein, thus making the two parts of the liver arise from separate germinal plates.

No reference is made to the function of the bile, so elaborately stated by Küss, viz., the desquamation and removal of the intestinal epithelium, and thus, by simply keeping the absorbents in order, *favoring* the absorption of fat, but not otherwise concerned in that work, though the absorption of fat has been considered the special rôle of the bile. The new theory seems reasonable, from the fact that the bile is not poured into the duodenum till some seven or eight hours after the ingestion of food, hence can have little or no part in digestion and absorption, as such.

The author treats of the kidneys as glandular organs and of the urine as a secretion of the same; whereas the purely mechanical pressure of the blood, the structure of the Malpighian corpuscles and the pathology of Bright's disease, to say nothing of the recent physiological experiments in this direction and the evidences from comparative anatomy, are very strong proofs that the kidneys are not glands in the strict physiological sense, but simply filters; that urea, in fact, no solid constituent of the urine, is secreted by peculiar gland cells in the kidney, but merely given up by drainage and passes out of the body as excrementitious matter, while the albumen so essential to nutrition is reabsorbed into the blood by the epithelium of the uriniferous tubuli, hence when the epithelium is diseased its function fails, and the albumen is left in the tubes to pass off in the urine. This to us is the most reasonable of all theories concerning the function of the kidneys—the only one that can account for all the phenomena.

The function of Peyer's patches is left quite obscure, although now apparently so well established — these organs being considered important centres of the lymphatic system, for the manufacture of white corpuscles; while the author gives an ambiguous assent to the formation of lymph corpuscles from the plasma—a modification of the spontaneous generative doctrine, recently revived by Onimus. We are surprised to see credence given to the exploded theory of Pavy, that the alkalinity of the blood protects the stomach from being digested by the gastric juice. We may well inquire, what then protects the intestine? There are many more plausible theories, such as "catalysis," the "active processes of nutrition" accepted by Flint, the coating of the stomach formed by mucus or desquamated epithelium during digestion, quoted by some authors, as Youmans in his last chemistry, but by far the most intelligent theory is the vital rôle of the epithelium of the part—of which a striking example is afforded by the epithelium of the urinary bladder.

The doubtful expression "coagulable lymph," is used, and the statement is made that the contractility of protoplasm is due to nervous influence. Whereas we all know contractility to be an inherent quality of protoplasm, which is manifest to a marked degree without the shadow of a nervous influence, unless we assume what it seems to us is true, that nervous force is only a part of the general force that characterizes living matter, that it is always active, but not recognized as such until it is set apart or differentiated from the common store and allowed to work through its own special organs.

We are glad to see the lymphoid organs, which include, as well, the so-called blood-vascular glands, have received so much consideration. Some of this ground has come to be no longer debatable, and yet most authors pass it over in silence. For example, Dalton's last work makes no mention of the spleen. It shows the true scientific spirit to attack the suprarenal capsules, the pituitary body, coccygeal gland and every atom that dares to find its way into the human body, put it into the witness box and make it speak for itself. We may be sure it will never bear false witness, the fault is in our interpretation. The only name we miss from this goodly company is that of the tonsils—physiology looks upon them no longer as sinecures.

The plates for the most part are very excellent, especially those of the nervous, absorbent and muscular systems, and those of the development of the embryo, though we do not accept some of the points of development, as for instance, that of the ovary. We look upon the epithelial element of the vesicles as an offshoot from the peritoneal epithelium, made by a dipping down into the stroma, of the investing peritoneal layer, these pouches finally becoming the closed and isolated vesicles.

Physiology is so far from being exact, every author who adds one atom to the side of certainty should receive the gratitude of us all—hence we welcome the present volume and heartily recommend it, save these few faults, to all, especially to those who can afford but one book on the subject. S. H. S.

L'Hygiène dans la Ville de Rome et dans La Campagne Romaine, par le Dr. Pietro Balestra, membre des Conseils Sanitaires Provinciaux, traduit de l'Italien. Paris. 1876. pp. 267.

If the traveler in Italy does not become disagreeably acquainted with the fever that prevails near the Roman Campagna, his guide-book will tell him about it. The student of malaria, too, discovers that this locality figures largely in the statistics of miasmatic disorders in Europe. A practical work on hygiene, for the benefit of those residing near the city of Rome, would therefore be valuable in many points of view, but the writer of this treatise has, we fear, failed to understand the purpose of such a handbook. The volume before us is, in many respects, too technical for the unprofessional reader, and yet is very far short of being a compendium of information respecting malarial disease. We suspect, cannot indeed avoid the suspicion, that the chief end in view of Dr. Balestra, was to increase his professional income by the publication, for the benefit of the laity, of a work which should contain sufficient scientific facts to impress the unprofessional reader with the extent of his learning.

"In order to do away with the sad results of an intermittent fever, we must avail ourselves as early as possible of the resources of art. I therefore advise the inhabitants of Rome to select a good physician," etc.

The author is inclined to adopt the view which would account for malaria, by the introduction into the system, of the spores of certain vegetable fungi. He quotes the article published in the *Chicago Medical Journal* for January 1874, by Dr. Jno. Bartlett, of this city, in corroboration of his views. But the name of our honorable colleague is distorted upon one page to Barlett, and upon another to Berlett. Let us charitably conclude that in the latter case only, has the author been in error.

